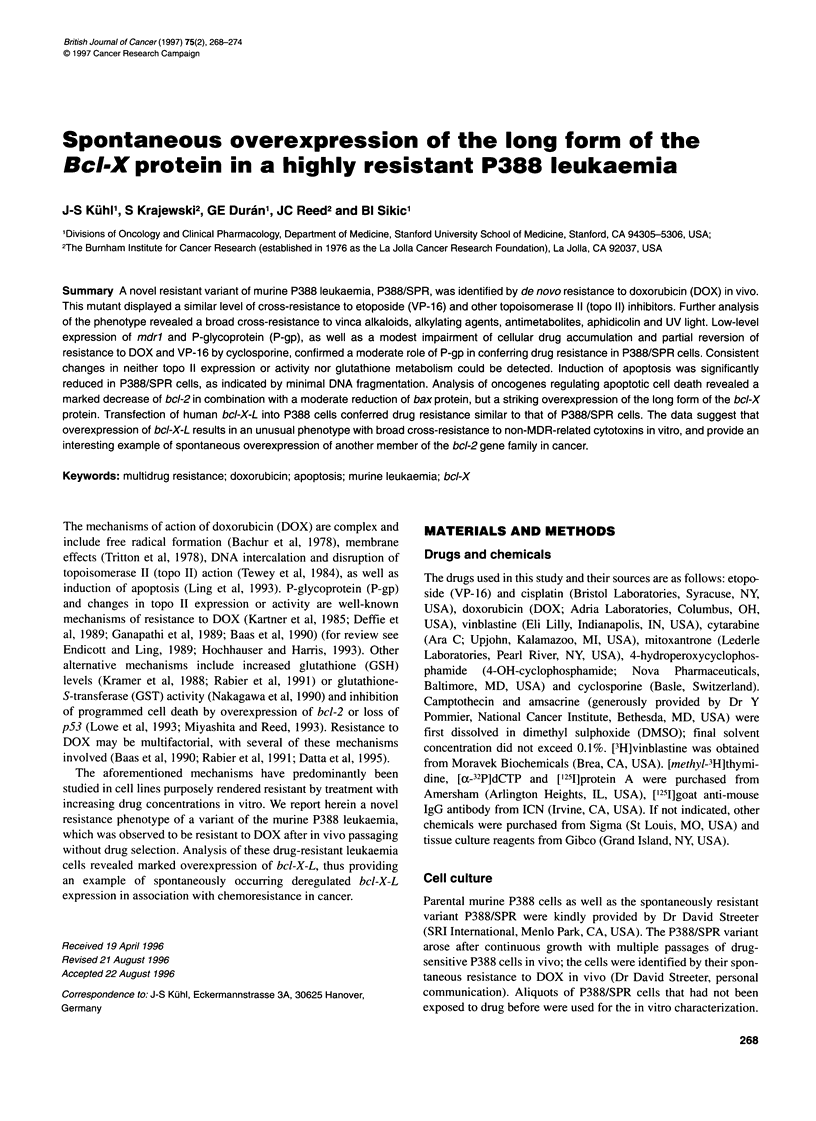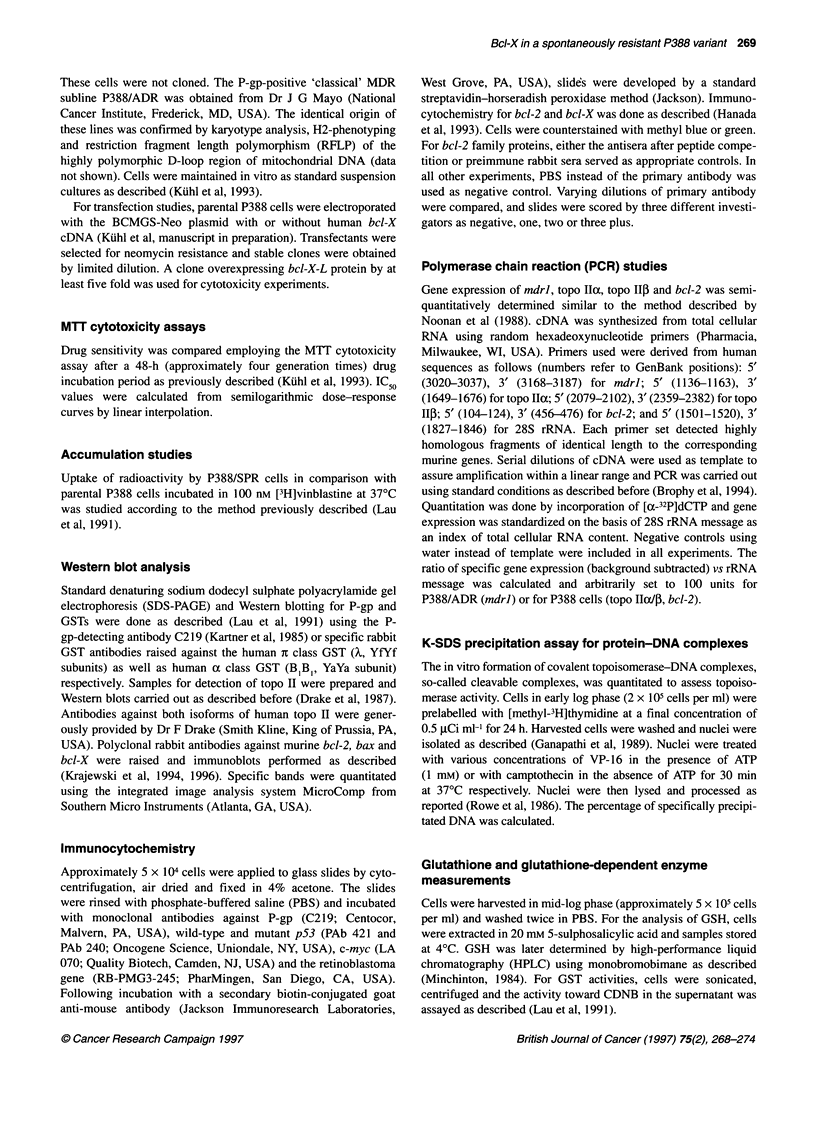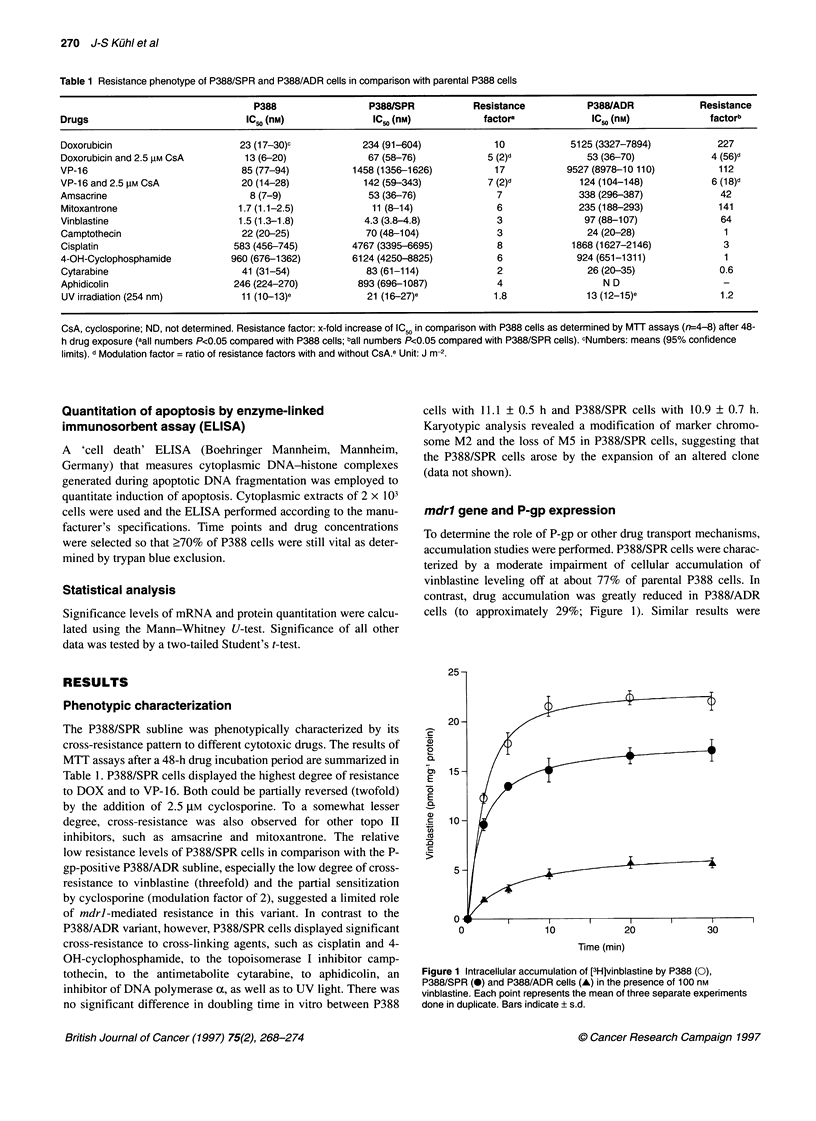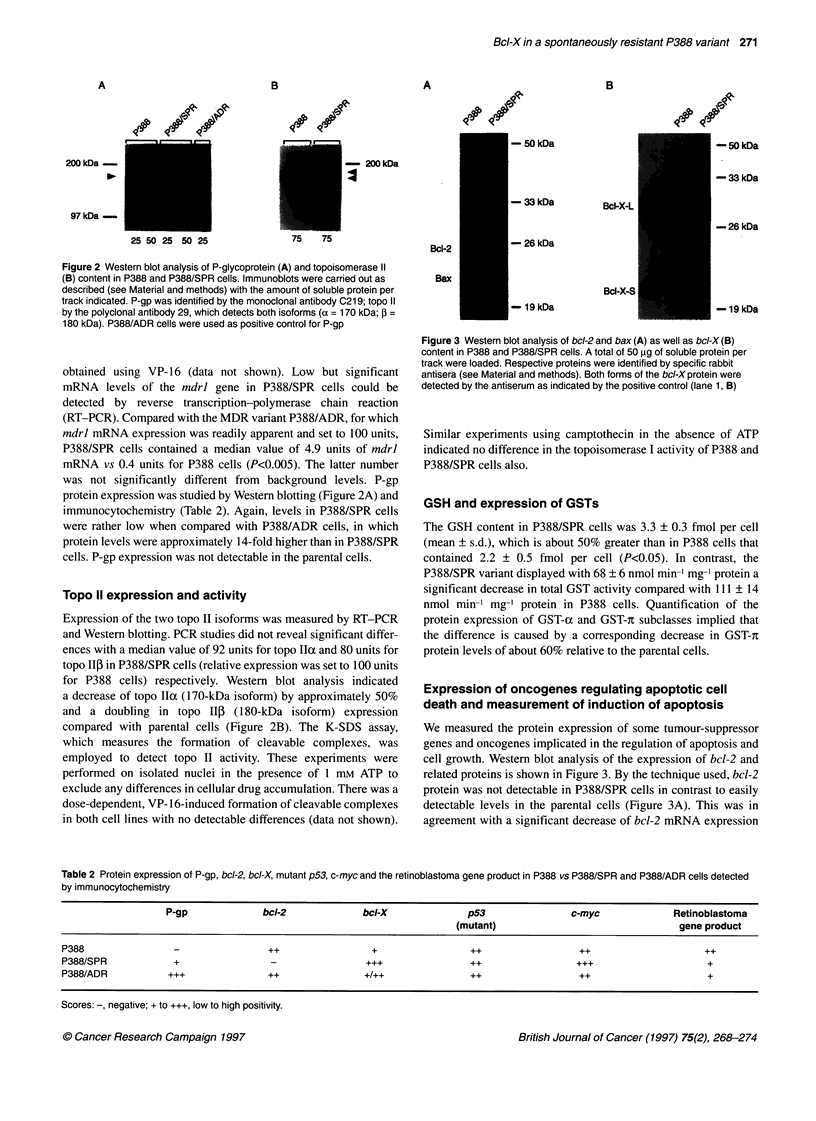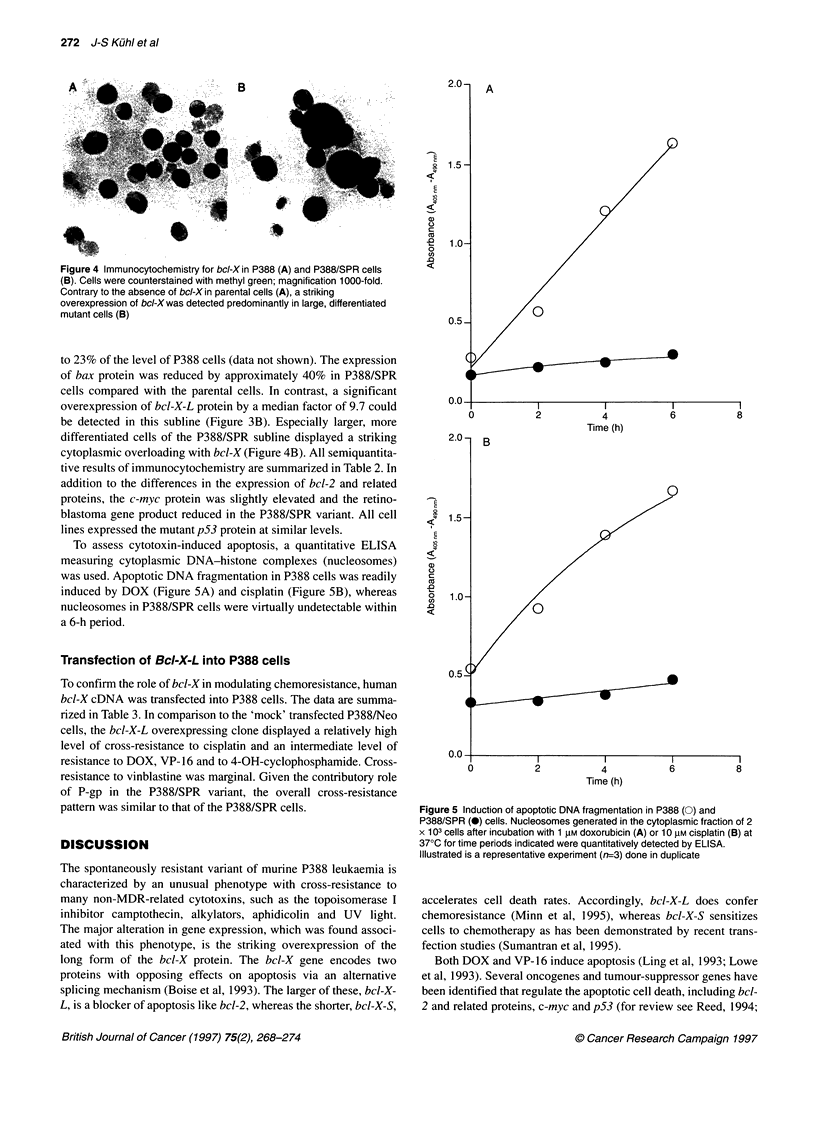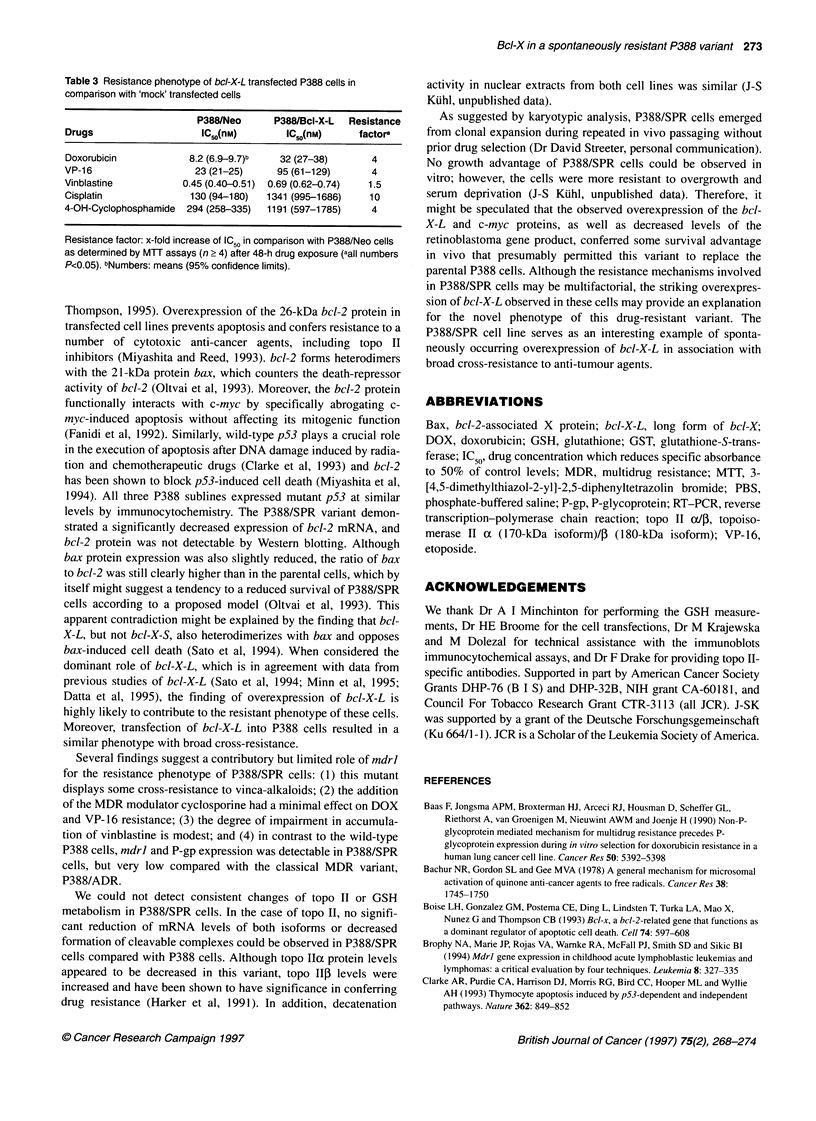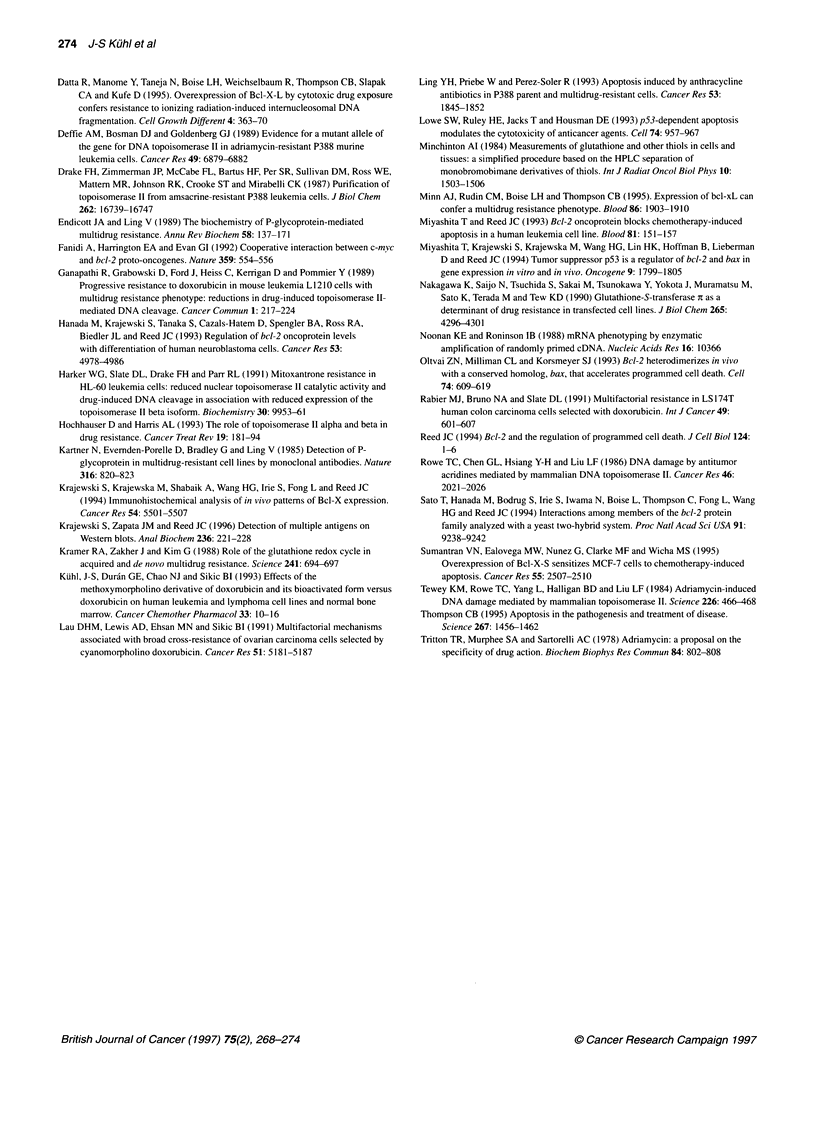# Spontaneous overexpression of the long form of the Bcl-X protein in a highly resistant P388 leukaemia.

**DOI:** 10.1038/bjc.1997.44

**Published:** 1997

**Authors:** J. S. Kühl, S. Krajewski, G. E. Durán, J. C. Reed, B. I. Sikic

**Affiliations:** Department of Medicine, Stanford University School of Medicine, CA 94305-5306, USA.

## Abstract

**Images:**


British Journal of Cancer (1997) 75(2), 268–274
© 1997 Cancer Research Campaign

### Spontaneous overexpression of the long form of the *Bcl-X* protein in a highly resistant P388 leukaemia

J-S Kühl[1], S Krajewski[2], GE Durán[1], JC Reed[2] and BI Sikic[1]

[1]Divisions of Oncology and Clinical Pharmacology, Department of Medicine, Stanford University School of Medicine, Stanford, CA 94305–5306, USA;
[2]The Burnham Institute for Cancer Research (established in 1976 as the La Jolla Cancer Research Foundation), La Jolla, CA 92037, USA

**Summary** A novel resistant variant of murine P388 leukaemia, P388/SPR, was identified by *de novo* resistance to doxorubicin (DOX) in vivo. This mutant displayed a similar level of cross-resistance to etoposide (VP-16) and other topoisomerase II (topo II) inhibitors. Further analysis of the phenotype revealed a broad cross-resistance to vinca alkaloids, alkylating agents, antimetabolites, aphidicolin and UV light. Low-level expression of *mdr1* and P-glycoprotein (P-gp), as well as a modest impairment of cellular drug accumulation and partial reversion of resistance to DOX and VP-16 by cyclosporine, confirmed a moderate role of P-gp in conferring drug resistance in P388/SPR cells. Consistent changes in neither topo II expression or activity nor glutathione metabolism could be detected. Induction of apoptosis was significantly reduced in P388/SPR cells, as indicated by minimal DNA fragmentation. Analysis of oncogenes regulating apoptotic cell death revealed a marked decrease of *bcl-2* in combination with a moderate reduction of *bax* protein, but a striking overexpression of the long form of the *bcl-X* protein. Transfection of human *bcl-X-L* into P388 cells conferred drug resistance similar to that of P388/SPR cells. The data suggest that overexpression of *bcl-X-L* results in an unusual phenotype with broad cross-resistance to non-MDR-related cytotoxins in vitro, and provide an interesting example of spontaneous overexpression of another member of the *bcl-2* gene family in cancer.

**Keywords:** multidrug resistance; doxorubicin; apoptosis; murine leukaemia; *bcl-X*

The mechanisms of action of doxorubicin (DOX) are complex and include free radical formation (Bachur et al, 1978), membrane effects (Tritton et al, 1978), DNA intercalation and disruption of topoisomerase II (topo II) action (Tewey et al, 1984), as well as induction of apoptosis (Ling et al, 1993). P-glycoprotein (P-gp) and changes in topo II expression or activity are well-known mechanisms of resistance to DOX (Kartner et al, 1985; Deffie et al, 1989; Ganapathi et al, 1989; Baas et al, 1990) (for review see Endicott and Ling, 1989; Hochhauser and Harris, 1993). Other alternative mechanisms include increased glutathione (GSH) levels (Kramer et al, 1988; Rabier et al, 1991) or glutathione-*S*-transferase (GST) activity (Nakagawa et al, 1990) and inhibition of programmed cell death by overexpression of *bcl-2* or loss of *p53* (Lowe et al, 1993; Miyashita and Reed, 1993). Resistance to DOX may be multifactorial, with several of these mechanisms involved (Baas et al, 1990; Rabier et al, 1991; Datta et al, 1995).

The aforementioned mechanisms have predominantly been studied in cell lines purposely rendered resistant by treatment with increasing drug concentrations in vitro. We report herein a novel resistance phenotype of a variant of the murine P388 leukaemia, which was observed to be resistant to DOX after in vivo passaging without drug selection. Analysis of these drug-resistant leukaemia cells revealed marked overexpression of *bcl-X-L*, thus providing an example of spontaneously occurring deregulated *bcl-X-L* expression in association with chemoresistance in cancer.

*Received 19 April 1996*
*Revised 21 August 1996*
*Accepted 22 August 1996*

*Correspondence to:* J-S Kühl, Eckermannstrasse 3A, 30625 Hanover, Germany

### MATERIALS AND METHODS

### Drugs and chemicals

The drugs used in this study and their sources are as follows: etoposide (VP-16) and cisplatin (Bristol Laboratories, Syracuse, NY, USA), doxorubicin (DOX; Adria Laboratories, Columbus, OH, USA), vinblastine (Eli Lilly, Indianapolis, IN, USA), cytarabine (Ara C; Upjohn, Kalamazoo, MI, USA), mitoxantrone (Lederle Laboratories, Pearl River, NY, USA), 4-hydroperoxycyclophosphamide (4-OH-cyclophosphamide; Nova Pharmaceuticals, Baltimore, MD, USA) and cyclosporine (Basle, Switzerland). Camptothecin and amsacrine (generously provided by Dr Y Pommier, National Cancer Institute, Bethesda, MD, USA) were first dissolved in dimethyl sulphoxide (DMSO); final solvent concentration did not exceed 0.1%. [$^3$H]vinblastine was obtained from Moravek Biochemicals (Brea, CA, USA). [*methyl*-$^3$H]thymidine, [α-$^{32}$P]dCTP and [$^{125}$I]protein A were purchased from Amersham (Arlington Heights, IL, USA), [$^{125}$I]goat anti-mouse IgG antibody from ICN (Irvine, CA, USA). If not indicated, other chemicals were purchased from Sigma (St Louis, MO, USA) and tissue culture reagents from Gibco (Grand Island, NY, USA).

### Cell culture

Parental murine P388 cells as well as the spontaneously resistant variant P388/SPR were kindly provided by Dr David Streeter (SRI International, Menlo Park, CA, USA). The P388/SPR variant arose after continuous growth with multiple passages of drug-sensitive P388 cells in vivo; the cells were identified by their spontaneous resistance to DOX in vivo (Dr David Streeter, personal communication). Aliquots of P388/SPR cells that had not been exposed to drug before were used for the in vitro characterization.

These cells were not cloned. The P-gp-positive 'classical' MDR subline P388/ADR was obtained from Dr J G Mayo (National Cancer Institute, Frederick, MD, USA). The identical origin of these lines was confirmed by karyotype analysis, H2-phenotyping and restriction fragment length polymorphism (RFLP) of the highly polymorphic D-loop region of mitochondrial DNA (data not shown). Cells were maintained in vitro as standard suspension cultures as described (Kühl et al, 1993).

For transfection studies, parental P388 cells were electroporated with the BCMGS-Neo plasmid with or without human *bcl-X* cDNA (Kühl et al, manuscript in preparation). Transfectants were selected for neomycin resistance and stable clones were obtained by limited dilution. A clone overexpressing *bcl-X-L* protein by at least five fold was used for cytotoxicity experiments.

### MTT cytotoxicity assays

Drug sensitivity was compared employing the MTT cytotoxicity assay after a 48-h (approximately four generation times) drug incubation period as previously described (Kühl et al, 1993). $IC_{50}$ values were calculated from semilogarithmic dose–response curves by linear interpolation.

### Accumulation studies

Uptake of radioactivity by P388/SPR cells in comparison with parental P388 cells incubated in 100 nM [$^3$H]vinblastine at 37°C was studied according to the method previously described (Lau et al, 1991).

### Western blot analysis

Standard denaturing sodium dodecyl sulphate polyacrylamide gel electrophoresis (SDS-PAGE) and Western blotting for P-gp and GSTs were done as described (Lau et al, 1991) using the P-gp-detecting antibody C219 (Kartner et al, 1985) or specific rabbit GST antibodies raised against the human π class GST (λ, YfYf subunits) as well as human α class GST ($B_1B_1$, YaYa subunit) respectively. Samples for detection of topo II were prepared and Western blots carried out as described before (Drake et al, 1987). Antibodies against both isoforms of human topo II were generously provided by Dr F Drake (Smith Kline, King of Prussia, PA, USA). Polyclonal rabbit antibodies against murine *bcl-2, bax* and *bcl-X* were raised and immunoblots performed as described (Krajewski et al, 1994, 1996). Specific bands were quantitated using the integrated image analysis system MicroComp from Southern Micro Instruments (Atlanta, GA, USA).

### Immunocytochemistry

Approximately $5 \times 10^4$ cells were applied to glass slides by cytocentrifugation, air dried and fixed in 4% acetone. The slides were rinsed with phosphate-buffered saline (PBS) and incubated with monoclonal antibodies against P-gp (C219; Centocor, Malvern, PA, USA), wild-type and mutant *p53* (PAb 421 and PAb 240; Oncogene Science, Uniondale, NY, USA), c-*myc* (LA 070; Quality Biotech, Camden, NJ, USA) and the retinoblastoma gene (RB-PMG3-245; PharMingen, San Diego, CA, USA). Following incubation with a secondary biotin-conjugated goat anti-mouse antibody (Jackson Immunoresearch Laboratories, West Grove, PA, USA), slides were developed by a standard streptavidin–horseradish peroxidase method (Jackson). Immunocytochemistry for *bcl-2* and *bcl-X* was done as described (Hanada et al, 1993). Cells were counterstained with methyl blue or green. For *bcl-2* family proteins, either the antisera after peptide competition or preimmune rabbit sera served as appropriate controls. In all other experiments, PBS instead of the primary antibody was used as negative control. Varying dilutions of primary antibody were compared, and slides were scored by three different investigators as negative, one, two or three plus.

### Polymerase chain reaction (PCR) studies

Gene expression of *mdr1*, topo IIα, topo IIβ and *bcl-2* was semi-quantitatively determined similar to the method described by Noonan et al (1988). cDNA was synthesized from total cellular RNA using random hexadeoxynucleotide primers (Pharmacia, Milwaukee, WI, USA). Primers used were derived from human sequences as follows (numbers refer to GenBank positions): 5′ (3020–3037), 3′ (3168–3187) for *mdr1*; 5′ (1136–1163), 3′ (1649–1676) for topo IIα; 5′ (2079–2102), 3′ (2359–2382) for topo IIβ; 5′ (104–124), 3′ (456–476) for *bcl-2*; and 5′ (1501–1520), 3′ (1827–1846) for 28S rRNA. Each primer set detected highly homologous fragments of identical length to the corresponding murine genes. Serial dilutions of cDNA were used as template to assure amplification within a linear range and PCR was carried out using standard conditions as described before (Brophy et al, 1994). Quantitation was done by incorporation of [α-$^{32}$P]dCTP and gene expression was standardized on the basis of 28S rRNA message as an index of total cellular RNA content. Negative controls using water instead of template were included in all experiments. The ratio of specific gene expression (background subtracted) *vs* rRNA message was calculated and arbitrarily set to 100 units for P388/ADR (*mdr1*) or for P388 cells (topo IIα/β, *bcl-2*).

### K-SDS precipitation assay for protein–DNA complexes

The in vitro formation of covalent topoisomerase–DNA complexes, so-called cleavable complexes, was quantitated to assess topoisomerase activity. Cells in early log phase ($2 \times 10^5$ cells per ml) were prelabelled with [methyl-$^3$H]thymidine at a final concentration of 0.5 μCi $ml^{-1}$ for 24 h. Harvested cells were washed and nuclei were isolated as described (Ganapathi et al, 1989). Nuclei were treated with various concentrations of VP-16 in the presence of ATP (1 mM) or with camptothecin in the absence of ATP for 30 min at 37°C respectively. Nuclei were then lysed and processed as reported (Rowe et al, 1986). The percentage of specifically precipitated DNA was calculated.

### Glutathione and glutathione-dependent enzyme measurements

Cells were harvested in mid-log phase (approximately $5 \times 10^5$ cells per ml) and washed twice in PBS. For the analysis of GSH, cells were extracted in 20 mM 5-sulphosalicylic acid and samples stored at 4°C. GSH was later determined by high-performance liquid chromatography (HPLC) using monobromobimane as described (Minchinton, 1984). For GST activities, cells were sonicated, centrifuged and the activity toward CDNB in the supernatant was assayed as described (Lau et al, 1991).

© Cancer Research Campaign 1997 

**Table 1** Resistance phenotype of P388/SPR and P388/ADR cells in comparison with parental P388 cells

| Drugs | P388 $IC_{50}$ (nM) | P388/SPR $IC_{50}$ (nM) | Resistance factor[a] | P388/ADR $IC_{50}$ (nM) | Resistance factor[b] |
|---|---|---|---|---|---|
| Doxorubicin | 23 (17–30)[c] | 234 (91–604) | 10 | 5125 (3327–7894) | 227 |
| Doxorubicin and 2.5 μM CsA | 13 (6–20) | 67 (58–76) | 5 (2)[d] | 53 (36–70) | 4 (56)[d] |
| VP-16 | 85 (77–94) | 1458 (1356–1626) | 17 | 9527 (8978–10 110) | 112 |
| VP-16 and 2.5 μM CsA | 20 (14–28) | 142 (59–343) | 7 (2)[d] | 124 (104–148) | 6 (18)[d] |
| Amsacrine | 8 (7–9) | 53 (36–76) | 7 | 338 (296–387) | 42 |
| Mitoxantrone | 1.7 (1.1–2.5) | 11 (8–14) | 6 | 235 (188–293) | 141 |
| Vinblastine | 1.5 (1.3–1.8) | 4.3 (3.8–4.8) | 3 | 97 (88–107) | 64 |
| Camptothecin | 22 (20–25) | 70 (48–104) | 3 | 24 (20–28) | 1 |
| Cisplatin | 583 (456–745) | 4767 (3395–6695) | 8 | 1868 (1627–2146) | 3 |
| 4-OH-Cyclophosphamide | 960 (676–1362) | 6124 (4250–8825) | 6 | 924 (651–1311) | 1 |
| Cytarabine | 41 (31–54) | 83 (61–114) | 2 | 26 (20–35) | 0.6 |
| Aphidicolin | 246 (224–270) | 893 (696–1087) | 4 | N D | – |
| UV irradiation (254 nm) | 11 (10–13)[e] | 21 (16–27)[e] | 1.8 | 13 (12–15)[e] | 1.2 |

CsA, cyclosporine; ND, not determined. Resistance factor: x-fold increase of $IC_{50}$ in comparison with P388 cells as determined by MTT assays ($n$=4–8) after 48-h drug exposure ([a]all numbers $P$<0.05 compared with P388 cells; [b]all numbers $P$<0.05 compared with P388/SPR cells). [c]Numbers: means (95% confidence limits). [d] Modulation factor = ratio of resistance factors with and without CsA.[e] Unit: J $m^{-2}$.

### Quantitation of apoptosis by enzyme-linked immunosorbent assay (ELISA)

A 'cell death' ELISA (Boehringer Mannheim, Mannheim, Germany) that measures cytoplasmic DNA–histone complexes generated during apoptotic DNA fragmentation was employed to quantitate induction of apoptosis. Cytoplasmic extracts of $2 \times 10^3$ cells were used and the ELISA performed according to the manufacturer's specifications. Time points and drug concentrations were selected so that ≥70% of P388 cells were still vital as determined by trypan blue exclusion.

### Statistical analysis

Significance levels of mRNA and protein quantitation were calculated using the Mann–Whitney $U$-test. Significance of all other data was tested by a two-tailed Student's $t$-test.

### RESULTS

### Phenotypic characterization

The P388/SPR subline was phenotypically characterized by its cross-resistance pattern to different cytotoxic drugs. The results of MTT assays after a 48-h drug incubation period are summarized in Table 1. P388/SPR cells displayed the highest degree of resistance to DOX and to VP-16. Both could be partially reversed (twofold) by the addition of 2.5 μM cyclosporine. To a somewhat lesser degree, cross-resistance was also observed for other topo II inhibitors, such as amsacrine and mitoxantrone. The relative low resistance levels of P388/SPR cells in comparison with the P-gp-positive P388/ADR subline, especially the low degree of cross-resistance to vinblastine (threefold) and the partial sensitization by cyclosporine (modulation factor of 2), suggested a limited role of *mdr1*-mediated resistance in this variant. In contrast to the P388/ADR variant, however, P388/SPR cells displayed significant cross-resistance to cross-linking agents, such as cisplatin and 4-OH-cyclophosphamide, to the topoisomerase I inhibitor camptothecin, to the antimetabolite cytarabine, to aphidicolin, an inhibitor of DNA polymerase α, as well as to UV light. There was no significant difference in doubling time in vitro between P388 cells with 11.1 ± 0.5 h and P388/SPR cells with 10.9 ± 0.7 h. Karyotypic analysis revealed a modification of marker chromosome M2 and the loss of M5 in P388/SPR cells, suggesting that the P388/SPR cells arose by the expansion of an altered clone (data not shown).

### *mdr1* gene and P-gp expression

To determine the role of P-gp or other drug transport mechanisms, accumulation studies were performed. P388/SPR cells were characterized by a moderate impairment of cellular accumulation of vinblastine leveling off at about 77% of parental P388 cells. In contrast, drug accumulation was greatly reduced in P388/ADR cells (to approximately 29%; Figure 1). Similar results were

**Figure 1** Intracellular accumulation of [$^3$H]vinblastine by P388 (○), P388/SPR (●) and P388/ADR cells (▲) in the presence of 100 nM vinblastine. Each point represents the mean of three separate experiments done in duplicate. Bars indicate ± s.d.

© Cancer Research Campaign 1997

**Figure 2** Western blot analysis of P-glycoprotein (**A**) and topoisomerase II (**B**) content in P388 and P388/SPR cells. Immunoblots were carried out as described (see Material and methods) with the amount of soluble protein per track indicated. P-gp was identified by the monoclonal antibody C219; topo II by the polyclonal antibody 29, which detects both isoforms ($\alpha$ = 170 kDa; $\beta$ = 180 kDa). P388/ADR cells were used as positive control for P-gp

**Figure 3** Western blot analysis of *bcl-2* and *bax* (**A**) as well as *bcl-X* (**B**) content in P388 and P388/SPR cells. A total of 50 µg of soluble protein per track were loaded. Respective proteins were identified by specific rabbit antisera (see Material and methods). Both forms of the *bcl-X* protein were detected by the antiserum as indicated by the positive control (lane 1, **B**)

obtained using VP-16 (data not shown). Low but significant mRNA levels of the *mdr1* gene in P388/SPR cells could be detected by reverse transcription–polymerase chain reaction (RT–PCR). Compared with the MDR variant P388/ADR, for which *mdr1* mRNA expression was readily apparent and set to 100 units, P388/SPR cells contained a median value of 4.9 units of *mdr1* mRNA *vs* 0.4 units for P388 cells ($P$<0.005). The latter number was not significantly different from background levels. P-gp protein expression was studied by Western blotting (Figure 2A) and immunocytochemistry (Table 2). Again, levels in P388/SPR cells were rather low when compared with P388/ADR cells, in which protein levels were approximately 14-fold higher than in P388/SPR cells. P-gp expression was not detectable in the parental cells.

### Topo II expression and activity

Expression of the two topo II isoforms was measured by RT–PCR and Western blotting. PCR studies did not reveal significant differences with a median value of 92 units for topo II$\alpha$ and 80 units for topo II$\beta$ in P388/SPR cells (relative expression was set to 100 units for P388 cells) respectively. Western blot analysis indicated a decrease of topo II$\alpha$ (170-kDa isoform) by approximately 50% and a doubling in topo II$\beta$ (180-kDa isoform) expression compared with parental cells (Figure 2B). The K-SDS assay, which measures the formation of cleavable complexes, was employed to detect topo II activity. These experiments were performed on isolated nuclei in the presence of 1 mM ATP to exclude any differences in cellular drug accumulation. There was a dose-dependent, VP-16-induced formation of cleavable complexes in both cell lines with no detectable differences (data not shown).

Similar experiments using camptothecin in the absence of ATP indicated no difference in the topoisomerase I activity of P388 and P388/SPR cells also.

### GSH and expression of GSTs

The GSH content in P388/SPR cells was 3.3 ± 0.3 fmol per cell (mean ± s.d.), which is about 50% greater than in P388 cells that contained 2.2 ± 0.5 fmol per cell ($P$<0.05). In contrast, the P388/SPR variant displayed with 68 ± 6 nmol $min^{-1}$ $mg^{-1}$ protein a significant decrease in total GST activity compared with 111 ± 14 nmol $min^{-1}$ $mg^{-1}$ protein in P388 cells. Quantification of the protein expression of GST-$\alpha$ and GST-$\pi$ subclasses implied that the difference is caused by a corresponding decrease in GST-$\pi$ protein levels of about 60% relative to the parental cells.

### Expression of oncogenes regulating apoptotic cell death and measurement of induction of apoptosis

We measured the protein expression of some tumour-suppressor genes and oncogenes implicated in the regulation of apoptosis and cell growth. Western blot analysis of the expression of *bcl-2* and related proteins is shown in Figure 3. By the technique used, *bcl-2* protein was not detectable in P388/SPR cells in contrast to easily detectable levels in the parental cells (Figure 3A). This was in agreement with a significant decrease of *bcl-2* mRNA expression

**Table 2** Protein expression of P-gp, *bcl-2*, *bcl-X*, mutant *p53*, c-*myc* and the retinoblastoma gene product in P388 *vs* P388/SPR and P388/ADR cells detected by immunocytochemistry

| | P-gp | *bcl-2* | *bcl-X* | *p53* (mutant) | *c-myc* | Retinoblastoma gene product |
|---|---|---|---|---|---|---|
| P388 | – | ++ | + | ++ | ++ | ++ |
| P388/SPR | + | – | +++ | ++ | +++ | + |
| P388/ADR | +++ | ++ | +/++ | ++ | ++ | + |

Scores: –, negative; + to +++, low to high positivity.

© Cancer Research Campaign 1997

**Figure 4** Immunocytochemistry for *bcl-X* in P388 (**A**) and P388/SPR cells (**B**). Cells were counterstained with methyl green; magnification 1000-fold. Contrary to the absence of *bcl-X* in parental cells (**A**), a striking overexpression of *bcl-X* was detected predominantly in large, differentiated mutant cells (**B**)

to 23% of the level of P388 cells (data not shown). The expression of *bax* protein was reduced by approximately 40% in P388/SPR cells compared with the parental cells. In contrast, a significant overexpression of *bcl-X-L* protein by a median factor of 9.7 could be detected in this subline (Figure 3B). Especially larger, more differentiated cells of the P388/SPR subline displayed a striking cytoplasmic overloading with *bcl-X* (Figure 4B). All semiquantitative results of immunocytochemistry are summarized in Table 2. In addition to the differences in the expression of *bcl-2* and related proteins, the *c-myc* protein was slightly elevated and the retinoblastoma gene product reduced in the P388/SPR variant. All cell lines expressed the mutant *p53* protein at similar levels.

To assess cytotoxin-induced apoptosis, a quantitative ELISA measuring cytoplasmic DNA–histone complexes (nucleosomes) was used. Apoptotic DNA fragmentation in P388 cells was readily induced by DOX (Figure 5A) and cisplatin (Figure 5B), whereas nucleosomes in P388/SPR cells were virtually undetectable within a 6-h period.

### Transfection of *Bcl-X-L* into P388 cells

To confirm the role of *bcl-X* in modulating chemoresistance, human *bcl-X* cDNA was transfected into P388 cells. The data are summarized in Table 3. In comparison to the 'mock' transfected P388/Neo cells, the *bcl-X-L* overexpressing clone displayed a relatively high level of cross-resistance to cisplatin and an intermediate level of resistance to DOX, VP-16 and to 4-OH-cyclophosphamide. Cross-resistance to vinblastine was marginal. Given the contributory role of P-gp in the P388/SPR variant, the overall cross-resistance pattern was similar to that of the P388/SPR cells.

### DISCUSSION

The spontaneously resistant variant of murine P388 leukaemia is characterized by an unusual phenotype with cross-resistance to many non-MDR-related cytotoxins, such as the topoisomerase I inhibitor camptothecin, alkylators, aphidicolin and UV light. The major alteration in gene expression, which was found associated with this phenotype, is the striking overexpression of the long form of the *bcl-X* protein. The *bcl-X* gene encodes two proteins with opposing effects on apoptosis via an alternative splicing mechanism (Boise et al, 1993). The larger of these, *bcl-X-L*, is a blocker of apoptosis like *bcl-2*, whereas the shorter, *bcl-X-S*,

**Figure 5** Induction of apoptotic DNA fragmentation in P388 (○) and P388/SPR (●) cells. Nucleosomes generated in the cytoplasmic fraction of 2 × $10^3$ cells after incubation with 1 μM doxorubicin (**A**) or 10 μM cisplatin (**B**) at 37°C for time periods indicated were quantitatively detected by ELISA. Illustrated is a representative experiment (*n*=3) done in duplicate accelerates cell death rates. Accordingly, *bcl-X-L* does confer chemoresistance (Minn et al, 1995), whereas *bcl-X-S* sensitizes cells to chemotherapy as has been demonstrated by recent transfection studies (Sumantran et al, 1995).

Both DOX and VP-16 induce apoptosis (Ling et al, 1993; Lowe et al, 1993). Several oncogenes and tumour-suppressor genes have been identified that regulate the apoptotic cell death, including *bcl-2* and related proteins, c-*myc* and *p53* (for review see Reed, 1994;

© Cancer Research Campaign 1997

**Table 3** Resistance phenotype of *bcl-X-L* transfected P388 cells in comparison with 'mock' transfected cells

| Drugs | P388/Neo $IC_{50}$(nM) | P388/Bcl-X-L $IC_{50}$(nM) | Resistance factor[a] |
|---|---|---|---|
| Doxorubicin | 8.2 (6.9–9.7)[b] | 32 (27–38) | 4 |
| VP-16 | 23 (21–25) | 95 (61–129) | 4 |
| Vinblastine | 0.45 (0.40–0.51) | 0.69 (0.62–0.74) | 1.5 |
| Cisplatin | 130 (94–180) | 1341 (995–1686) | 10 |
| 4-OH-Cyclophosphamide | 294 (258–335) | 1191 (597–1785) | 4 |

Resistance factor: x-fold increase of $IC_{50}$ in comparison with P388/Neo cells as determined by MTT assays ($n \geq 4$) after 48-h drug exposure ([a]all numbers $P<0.05$). [b]Numbers: means (95% confidence limits).

Thompson, 1995). Overexpression of the 26-kDa *bcl-2* protein in transfected cell lines prevents apoptosis and confers resistance to a number of cytotoxic anti-cancer agents, including topo II inhibitors (Miyashita and Reed, 1993). *bcl-2* forms heterodimers with the 21-kDa protein *bax*, which counters the death-repressor activity of *bcl-2* (Oltvai et al, 1993). Moreover, the *bcl-2* protein functionally interacts with c-*myc* by specifically abrogating c-*myc*-induced apoptosis without affecting its mitogenic function (Fanidi et al, 1992). Similarly, wild-type *p53* plays a crucial role in the execution of apoptosis after DNA damage induced by radiation and chemotherapeutic drugs (Clarke et al, 1993) and *bcl-2* has been shown to block *p53*-induced cell death (Miyashita et al, 1994). All three P388 sublines expressed mutant *p53* at similar levels by immunocytochemistry. The P388/SPR variant demonstrated a significantly decreased expression of *bcl-2* mRNA, and *bcl-2* protein was not detectable by Western blotting. Although *bax* protein expression was also slightly reduced, the ratio of *bax* to *bcl-2* was still clearly higher than in the parental cells, which by itself might suggest a tendency to a reduced survival of P388/SPR cells according to a proposed model (Oltvai et al, 1993). This apparent contradiction might be explained by the finding that *bcl-X-L*, but not *bcl-X-S*, also heterodimerizes with *bax* and opposes *bax*-induced cell death (Sato et al, 1994). When considered the dominant role of *bcl-X-L*, which is in agreement with data from previous studies of *bcl-X-L* (Sato et al, 1994; Minn et al, 1995; Datta et al, 1995), the finding of overexpression of *bcl-X-L* is highly likely to contribute to the resistant phenotype of these cells. Moreover, transfection of *bcl-X-L* into P388 cells resulted in a similar phenotype with broad cross-resistance.

Several findings suggest a contributory but limited role of *mdr1* for the resistance phenotype of P388/SPR cells: (1) this mutant displays some cross-resistance to vinca-alkaloids; (2) the addition of the MDR modulator cyclosporine had a minimal effect on DOX and VP-16 resistance; (3) the degree of impairment in accumulation of vinblastine is modest; and (4) in contrast to the wild-type P388 cells, *mdr1* and P-gp expression was detectable in P388/SPR cells, but very low compared with the classical MDR variant, P388/ADR.

We could not detect consistent changes of topo II or GSH metabolism in P388/SPR cells. In the case of topo II, no significant reduction of mRNA levels of both isoforms or decreased formation of cleavable complexes could be observed in P388/SPR cells compared with P388 cells. Although topo II$\alpha$ protein levels appeared to be decreased in this variant, topo II$\beta$ levels were increased and have been shown to have significance in conferring drug resistance (Harker et al, 1991). In addition, decatenation activity in nuclear extracts from both cell lines was similar (J-S Kühl, unpublished data).

As suggested by karyotypic analysis, P388/SPR cells emerged from clonal expansion during repeated in vivo passaging without prior drug selection (Dr David Streeter, personal communication). No growth advantage of P388/SPR cells could be observed in vitro; however, the cells were more resistant to overgrowth and serum deprivation (J-S Kühl, unpublished data). Therefore, it might be speculated that the observed overexpression of the *bcl-X-L* and c-*myc* proteins, as well as decreased levels of the retinoblastoma gene product, conferred some survival advantage in vivo that presumably permitted this variant to replace the parental P388 cells. Although the resistance mechanisms involved in P388/SPR cells may be multifactorial, the striking overexpression of *bcl-X-L* observed in these cells may provide an explanation for the novel phenotype of this drug-resistant variant. The P388/SPR cell line serves as an interesting example of spontaneously occurring overexpression of *bcl-X-L* in association with broad cross-resistance to anti-tumour agents.

### ABBREVIATIONS

Bax, *bcl-2*-associated X protein; *bcl-X-L*, long form of *bcl-X*; DOX, doxorubicin; GSH, glutathione; GST, glutathione-*S*-transferase; $IC_{50}$, drug concentration which reduces specific absorbance to 50% of control levels; MDR, multidrug resistance; MTT, 3-[4,5-dimethylthiazol-2-yl]-2,5-diphenyltetrazolin bromide; PBS, phosphate-buffered saline; P-gp, P-glycoprotein; RT–PCR, reverse transcription–polymerase chain reaction; topo II $\alpha/\beta$, topoisomerase II $\alpha$ (170-kDa isoform)/$\beta$ (180-kDa isoform); VP-16, etoposide.

### ACKNOWLEDGEMENTS

We thank Dr A I Minchinton for performing the GSH measurements, Dr HE Broome for the cell transfections, Dr M Krajewska and M Dolezal for technical assistance with the immunoblots immunocytochemical assays, and Dr F Drake for providing topo II-specific antibodies. Supported in part by American Cancer Society Grants DHP-76 (B I S) and DHP-32B, NIH grant CA-60181, and Council For Tobacco Research Grant CTR-3113 (all JCR). J-SK was supported by a grant of the Deutsche Forschungsgemeinschaft (Ku 664/1-1). JCR is a Scholar of the Leukemia Society of America.

### REFERENCES

Baas F, Jongsma APM, Broxterman HJ, Arceci RJ, Housman D, Scheffer GL, Riethorst A, van Groenigen M, Nieuwint AWM and Joenje H (1990) Non-P-glycoprotein mediated mechanism for multidrug resistance precedes P-glycoprotein expression during *in vitro* selection for doxorubicin resistance in a human lung cancer cell line. *Cancer Res* **50**: 5392–5398

Bachur NR, Gordon SL and Gee MVA (1978) A general mechanism for microsomal activation of quinone anti-cancer agents to free radicals. *Cancer Res* **38**: 1745–1750

Boise LH, Gonzalez GM, Postema CE, Ding L, Lindsten T, Turka LA, Mao X, Nunez G and Thompson CB (1993) *Bcl-x*, a *bcl-2*-related gene that functions as a dominant regulator of apoptotic cell death. *Cell* **74**: 597–608

Brophy NA, Marie JP, Rojas VA, Warnke RA, McFall PJ, Smith SD and Sikic BI (1994) *Mdr1* gene expression in childhood acute lymphoblastic leukemias and lymphomas: a critical evaluation by four techniques. *Leukemia* **8**: 327–335

Clarke AR, Purdie CA, Harrison DJ, Morris RG, Bird CC, Hooper ML and Wyllie AH (1993) Thymocyte apoptosis induced by *p53*-dependent and independent pathways. *Nature* **362**: 849–852

© Cancer Research Campaign 1997 

Datta R, Manome Y, Taneja N, Boise LH, Weichselbaum R, Thompson CB, Slapak CA and Kufe D (1995). Overexpression of Bcl-X-L by cytotoxic drug exposure confers resistance to ionizing radiation-induced internucleosomal DNA fragmentation. *Cell Growth Different* **4**: 363–70

Deffie AM, Bosman DJ and Goldenberg GJ (1989) Evidence for a mutant allele of the gene for DNA topoisomerase II in adriamycin-resistant P388 murine leukemia cells. *Cancer Res* **49**: 6879–6882

Drake FH, Zimmerman JP, McCabe FL, Bartus HF, Per SR, Sullivan DM, Ross WE, Mattern MR, Johnson RK, Crooke ST and Mirabelli CK (1987) Purification of topoisomerase II from amsacrine-resistant P388 leukemia cells. *J Biol Chem* **262**: 16739–16747

Endicott JA and Ling V (1989) The biochemistry of P-glycoprotein-mediated multidrug resistance. *Annu Rev Biochem* **58**: 137–171

Fanidi A, Harrington EA and Evan GI (1992) Cooperative interaction between c-*myc* and *bcl-2* proto-oncogenes. *Nature* **359**: 554–556

Ganapathi R, Grabowski D, Ford J, Heiss C, Kerrigan D and Pommier Y (1989) Progressive resistance to doxorubicin in mouse leukemia L1210 cells with multidrug resistance phenotype: reductions in drug-induced topoisomerase II-mediated DNA cleavage. *Cancer Commun* **1**: 217–224

Hanada M, Krajewski S, Tanaka S, Cazals-Hatem D, Spengler BA, Ross RA, Biedler JL and Reed JC (1993) Regulation of *bcl-2* oncoprotein levels with differentiation of human neuroblastoma cells. *Cancer Res* **53**: 4978–4986

Harker WG, Slate DL, Drake FH and Parr RL (1991) Mitoxantrone resistance in HL-60 leukemia cells: reduced nuclear topoisomerase II catalytic activity and drug-induced DNA cleavage in association with reduced expression of the topoisomerase II beta isoform. *Biochemistry* **30**: 9953–61

Hochhauser D and Harris AL (1993) The role of topoisomerase II alpha and beta in drug resistance. *Cancer Treat Rev* **19**: 181–94

Kartner N, Evernden-Porelle D, Bradley G and Ling V (1985) Detection of P-glycoprotein in multidrug-resistant cell lines by monoclonal antibodies. *Nature* **316**: 820–823

Krajewski S, Krajewska M, Shabaik A, Wang HG, Irie S, Fong L and Reed JC (1994) Immunohistochemical analysis of *in vivo* patterns of Bcl-X expression. *Cancer Res* **54**: 5501–5507

Krajewski S, Zapata JM and Reed JC (1996) Detection of multiple antigens on Western blots. *Anal Biochem* **236**: 221–228

Kramer RA, Zakher J and Kim G (1988) Role of the glutathione redox cycle in acquired and *de novo* multidrug resistance. *Science* **241**: 694–697

Kühl, J-S, Durán GE, Chao NJ and Sikic BI (1993) Effects of the methoxymorpholino derivative of doxorubicin and its bioactivated form versus doxorubicin on human leukemia and lymphoma cell lines and normal bone marrow. *Cancer Chemother Pharmacol* **33**: 10–16

Lau DHM, Lewis AD, Ehsan MN and Sikic BI (1991) Multifactorial mechanisms associated with broad cross-resistance of ovarian carcinoma cells selected by cyanomorpholino doxorubicin. *Cancer Res* **51**: 5181–5187

Ling YH, Priebe W and Perez-Soler R (1993) Apoptosis induced by anthracycline antibiotics in P388 parent and multidrug-resistant cells. *Cancer Res* **53**: 1845–1852

Lowe SW, Ruley HE, Jacks T and Housman DE (1993) *p53*-dependent apoptosis modulates the cytotoxicity of anticancer agents. *Cell* **74**: 957–967

Minchinton AI (1984) Measurements of glutathione and other thiols in cells and tissues: a simplified procedure based on the HPLC separation of monobromobimane derivatives of thiols. *Int J Radiat Oncol Biol Phys* **10**: 1503–1506

Minn AJ, Rudin CM, Boise LH and Thompson CB (1995). Expression of bcl-xL can confer a multidrug resistance phenotype. *Blood* **86**: 1903–1910

Miyashita T and Reed JC (1993) *Bcl-2* oncoprotein blocks chemotherapy-induced apoptosis in a human leukemia cell line. *Blood* **81**: 151–157

Miyashita T, Krajewski S, Krajewska M, Wang HG, Lin HK, Hoffman B, Lieberman D and Reed JC (1994) Tumor suppressor p53 is a regulator of *bcl-2* and *bax* in gene expression *in vitro* and *in vivo*. *Oncogene* **9**: 1799–1805

Nakagawa K, Saijo N, Tsuchida S, Sakai M, Tsunokawa Y, Yokota J, Muramatsu M, Sato K, Terada M and Tew KD (1990) Glutathione-*S*-transferase $\pi$ as a determinant of drug resistance in transfected cell lines. *J Biol Chem* **265**: 4296–4301

Noonan KE and Roninson IB (1988) mRNA phenotyping by enzymatic amplification of randomly primed cDNA. *Nucleic Acids Res* **16**: 10366

Oltvai ZN, Milliman CL and Korsmeyer SJ (1993) *Bcl-2* heterodimerizes *in vivo* with a conserved homolog, *bax*, that accelerates programmed cell death. *Cell* **74**: 609–619

Rabier MJ, Bruno NA and Slate DL (1991) Multifactorial resistance in LS174T human colon carcinoma cells selected with doxorubicin. *Int J Cancer* **49**: 601–607

Reed JC (1994) *Bcl-2* and the regulation of programmed cell death. *J Cell Biol* **124**: 1–6

Rowe TC, Chen GL, Hsiang Y-H and Liu LF (1986) DNA damage by antitumor acridines mediated by mammalian DNA topoisomerase II. *Cancer Res* **46**: 2021–2026

Sato T, Hanada M, Bodrug S, Irie S, Iwama N, Boise L, Thompson C, Fong L, Wang HG and Reed JC (1994) Interactions among members of the *bcl-2* protein family analyzed with a yeast two-hybrid system. *Proc Natl Acad Sci USA* **91**: 9238–9242

Sumantran VN, Ealovega MW, Nunez G, Clarke MF and Wicha MS (1995) Overexpression of Bcl-X-S sensitizes MCF-7 cells to chemotherapy-induced apoptosis. *Cancer Res* **55**: 2507–2510

Tewey KM, Rowe TC, Yang L, Halligan BD and Liu LF (1984) Adriamycin-induced DNA damage mediated by mammalian topoisomerase II. *Science* **226**: 466–468

Thompson CB (1995) Apoptosis in the pathogenesis and treatment of disease. *Science* **267**: 1456–1462

Tritton TR, Murphee SA and Sartorelli AC (1978) Adriamycin: a proposal on the specificity of drug action. *Biochem Biophys Res Commun* **84**: 802–808

 *© Cancer Research Campaign 1997*